# Supplementary figures and images for: The proximal proteome of 17 SARS-CoV-2 proteins links to disrupted antiviral signaling and host translation
Source: PLoS Pathog. 2021 Oct 1;17(10):e1009412. doi: 10.1371/journal.ppat.1009412 (PMC8513853; doi:10.1371/journal.ppat.1009412)

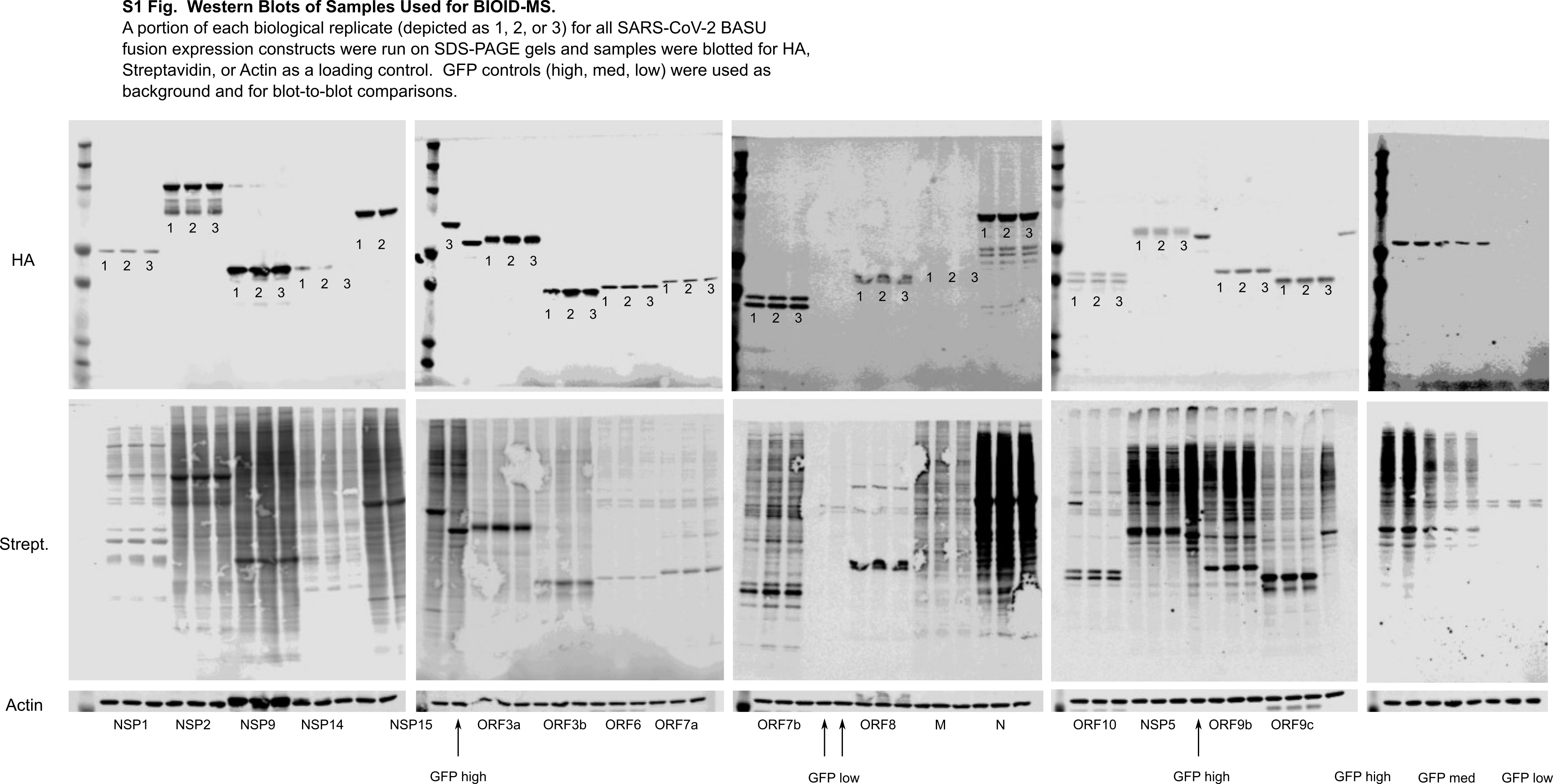

Supplement: S1 Fig — A portion of each biological replicate (depicted as 1, 2, or 3) for all SARS-CoV-2 BASU fusion expression constructs were run on SDS-PAGE gels and samples were blotted for HA, Streptavidin, or Actin as a loading control. GFP controls (high, med, low) were used as background and for blot-to-blot comparisons. (TIF) [file ppat.1009412.s001.tif]

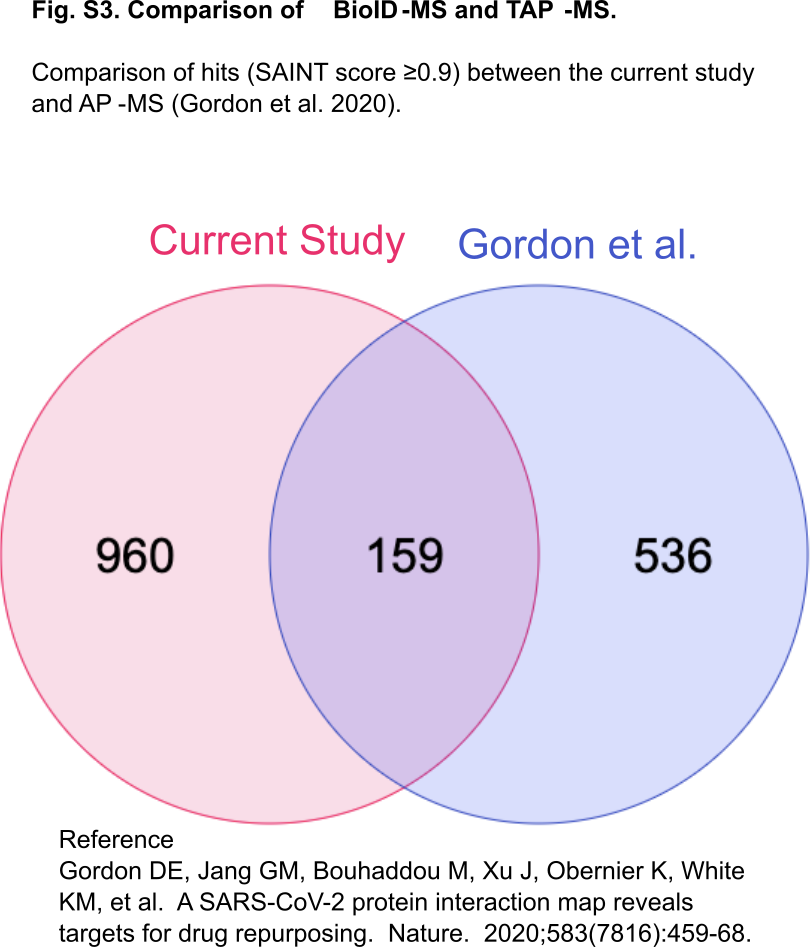

Supplement: S3 Fig — Comparison of hits (SAINT score ≥0.9) between the current study and AP-MS (Gordon et al. 2020). (TIF) [file ppat.1009412.s003.tif]

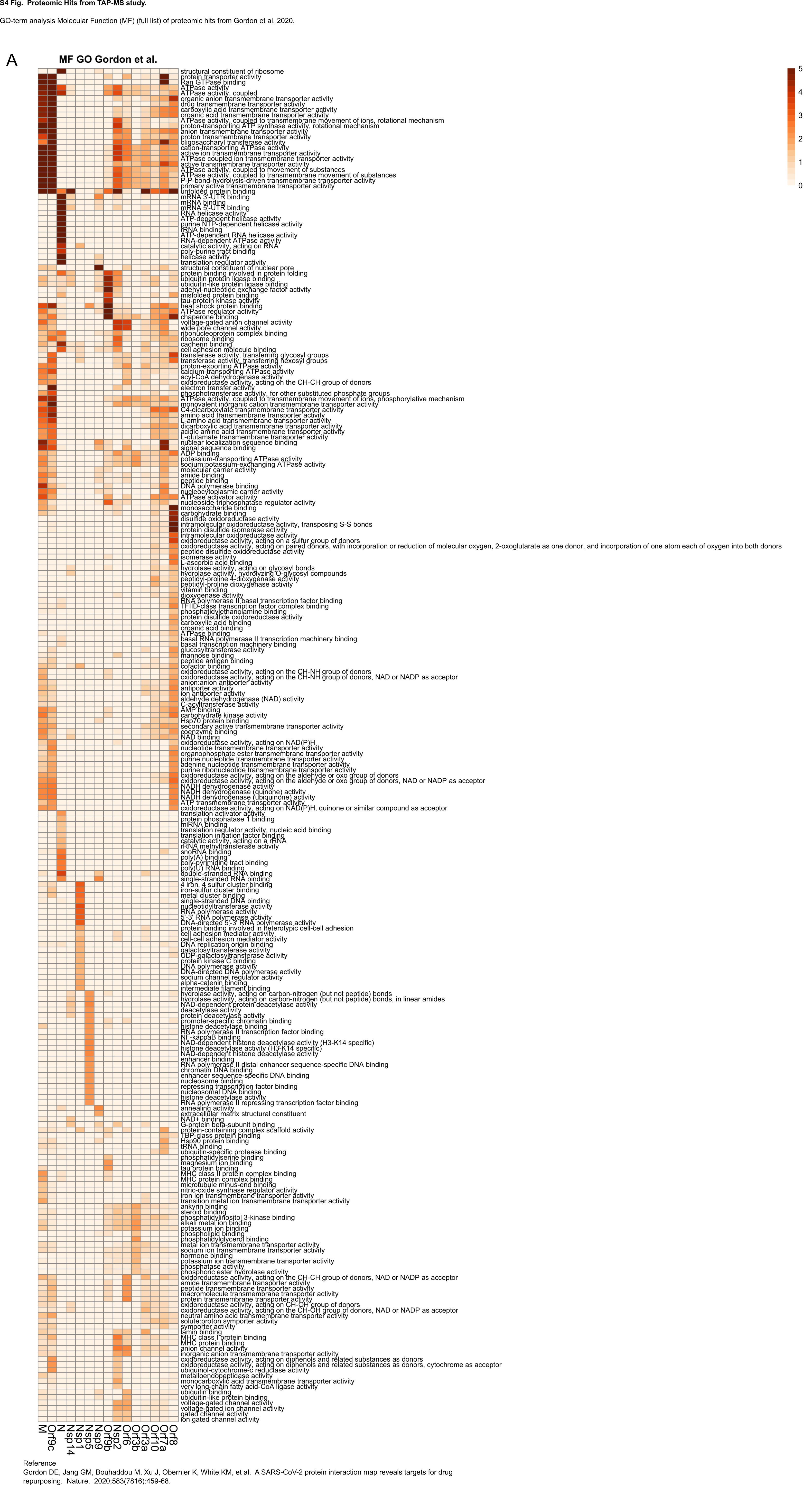

Supplement: S4 Fig — GO-term analysis Molecular Function (MF) (full list) of proteomic hits from Gordon et al. 2020. (TIF) [file ppat.1009412.s004.tif]

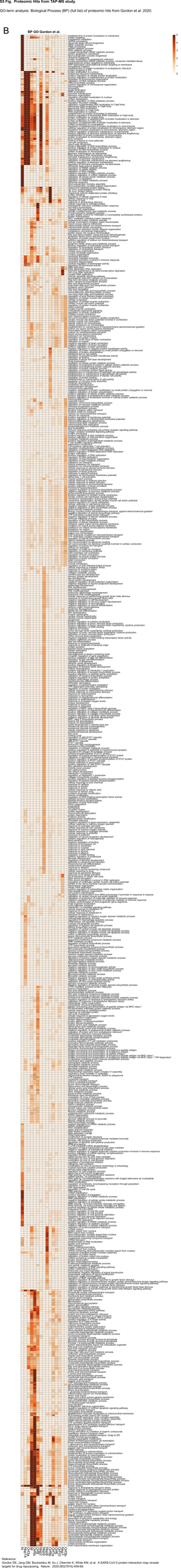

Supplement: S5 Fig — GO-term analysis Biological Processes (BP) (full list) of proteomic hits from Gordon et al. 2020. (TIF) [file ppat.1009412.s005.tif]

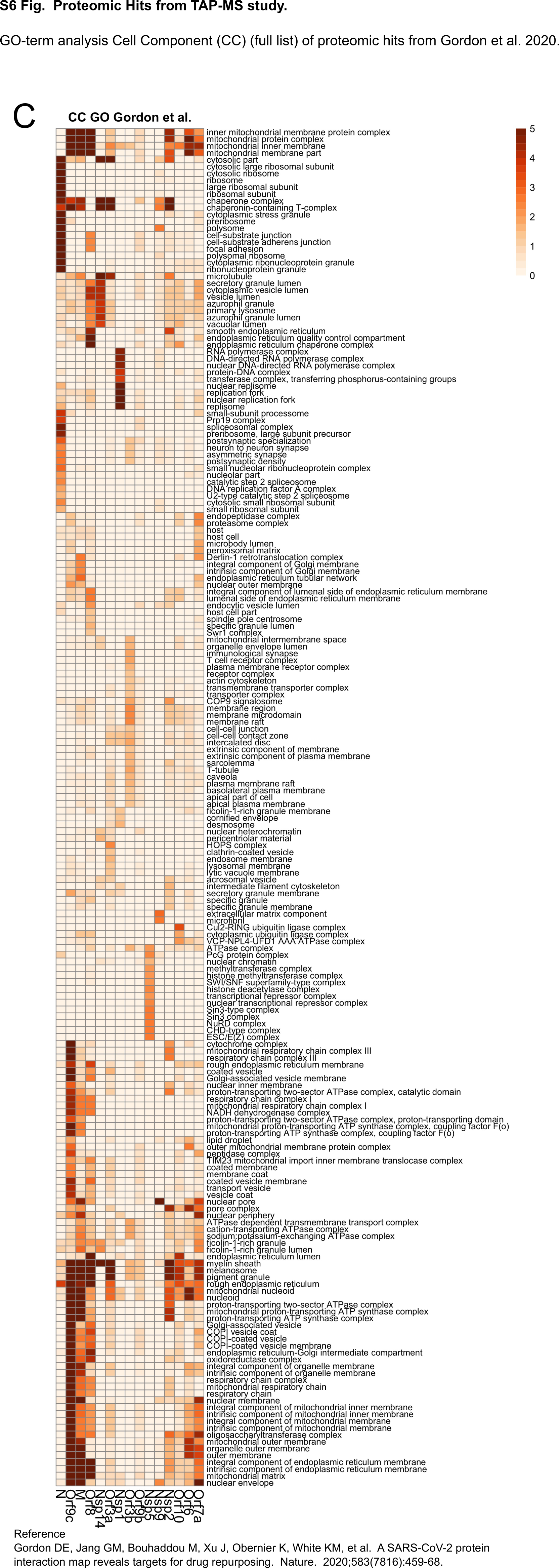

Supplement: S6 Fig — GO-term analysis Cell Component (CC) (full list) of proteomic hits from Gordon et al. 2020. (TIF) [file ppat.1009412.s006.tif]

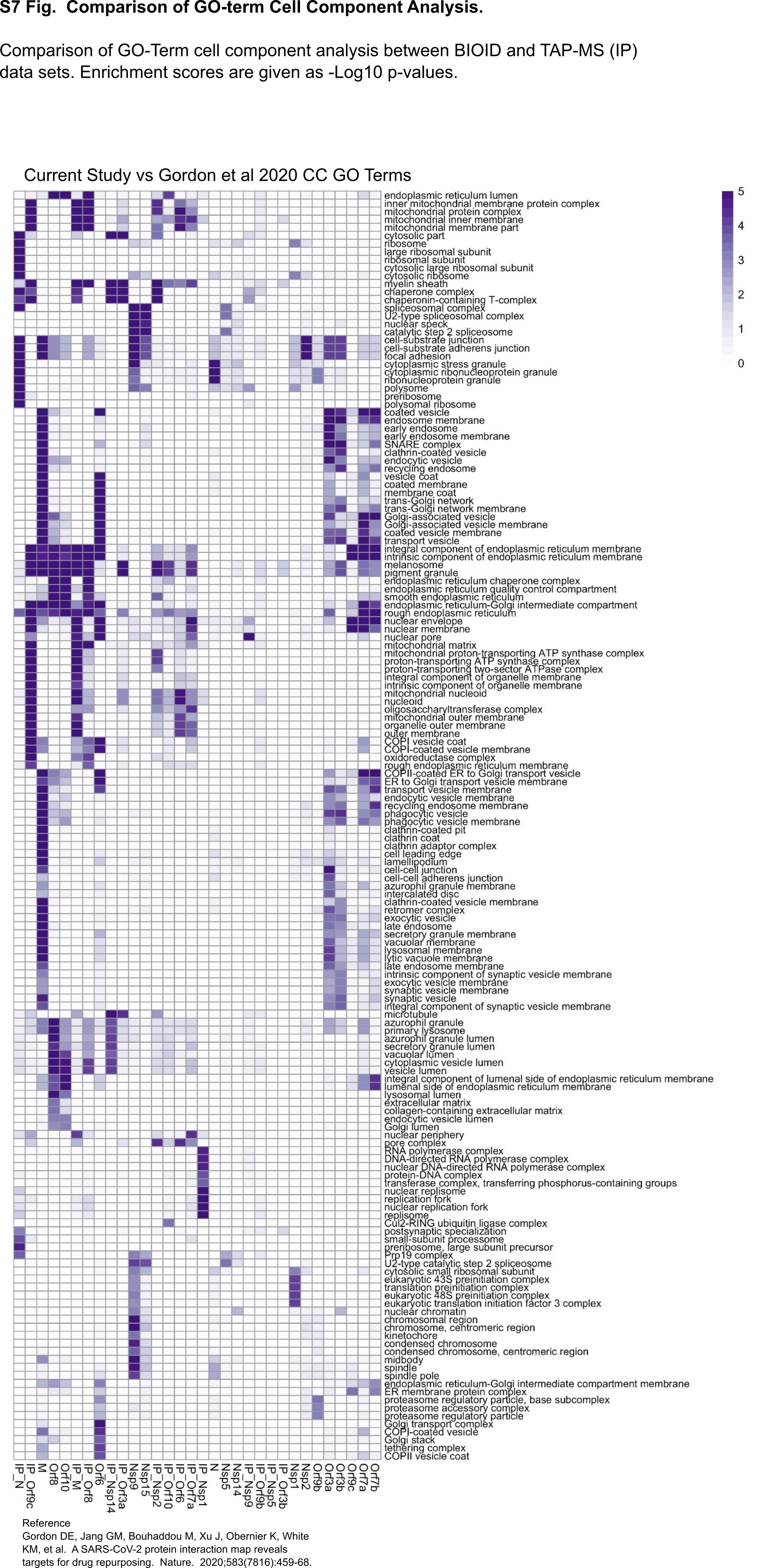

Supplement: S7 Fig — Comparison of cell component GO-term analysis between BIOID and TAP-MS (IP) data sets. Enrichment scores are given as -Log10 p-values. (TIF) [file ppat.1009412.s007.tif]

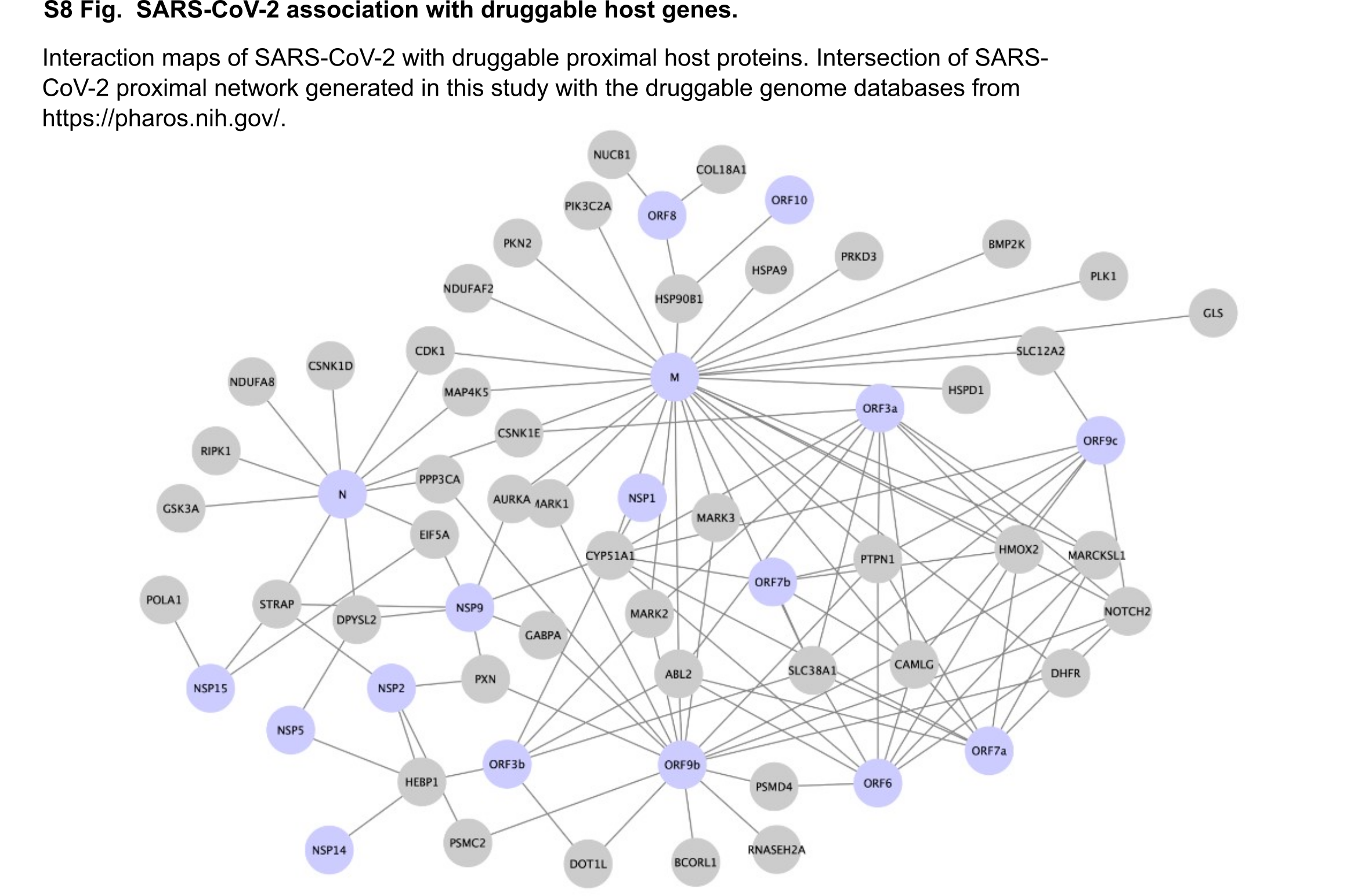

Supplement: S8 Fig — Interaction maps of SARS-CoV-2 with druggable proximal host proteins. Intersection of SARS-CoV-2 proximal network generated in this study with the druggable genome databases from https://pharos.nih.gov/. (TIF) [file ppat.1009412.s008.tif]

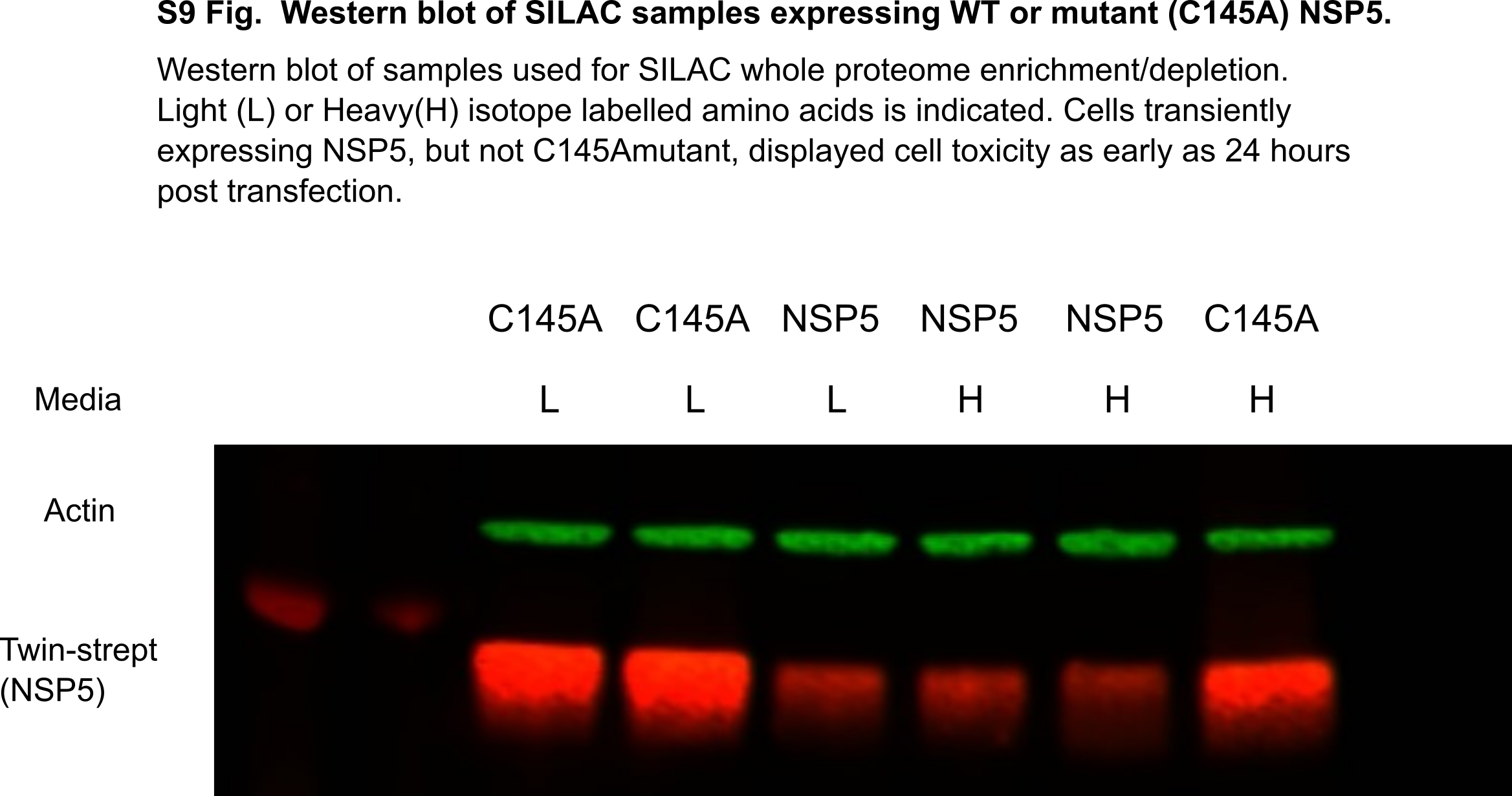

Supplement: S9 Fig — Western blot of samples used for SILAC whole proteome enrichment/depletion. Light (L) or Heavy (H) isotope labelled amino acids is indicated. Cells transiently expressing NSP5, but not C145A mutant, displayed cell toxicity as early as 24 hours post transfection. (TIF) [file ppat.1009412.s009.tif]
