# Supplementary material for: The proximal proteome of 17 SARS-CoV-2 proteins links to disrupted antiviral signaling and host translation
Source: PLoS Pathog. 2021 Oct 1;17(10):e1009412. doi: 10.1371/journal.ppat.1009412 (PMC8513853; doi:10.1371/journal.ppat.1009412)

S2 Fig. Interaction Maps for SARS-CoV-2 proteins

Interaction maps for all SARS-CoV-2 proteins(A-Q) from this study. All host proteins with SAINT scores ≥ 0.9 were included.

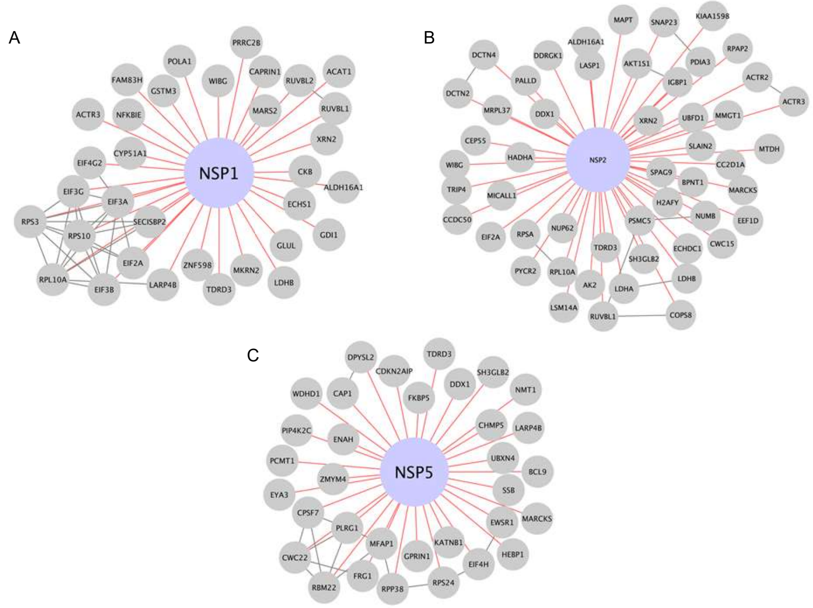

S2 Fig. Interaction maps of SARS-CoV-2 proteins (cont'd)

D

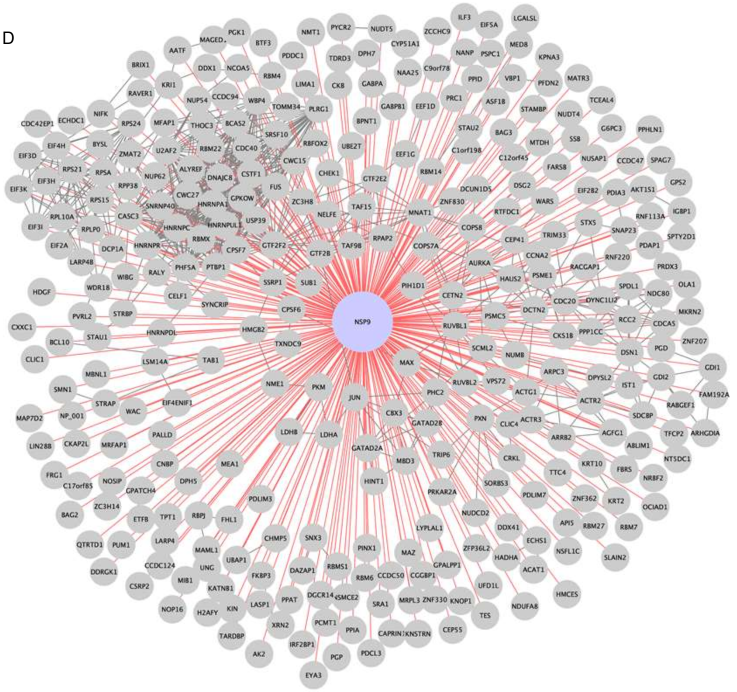

S2 Fig. Interaction maps of SARS-CoV-2 proteins (cont'd)

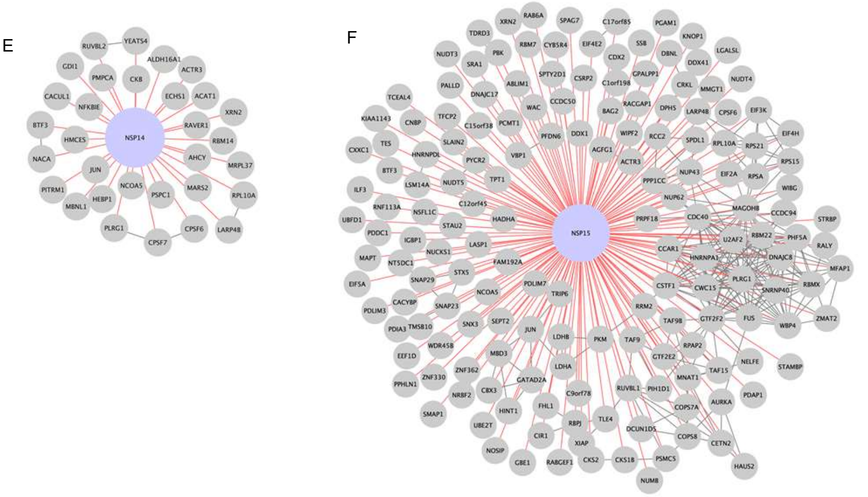

S2 Fig. Interaction maps of SARS-CoV-2 proteins (cont'd)

G

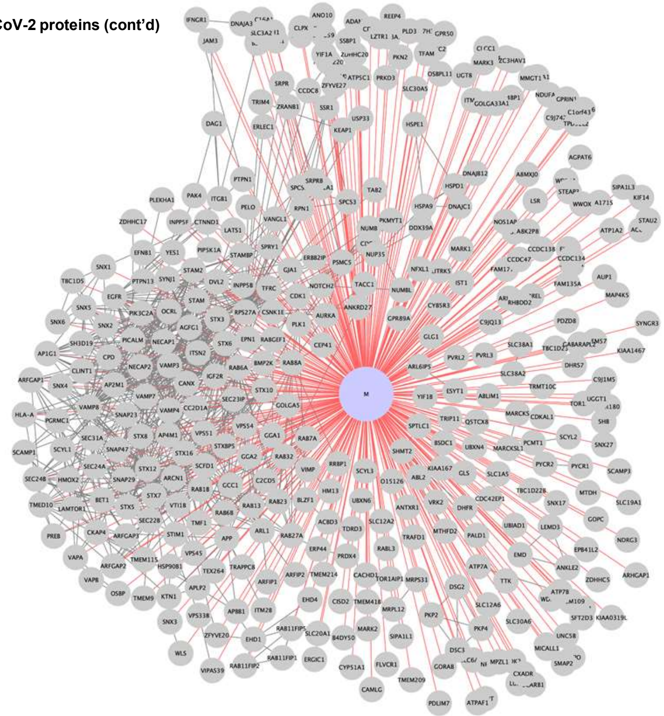

S2 Fig. Interaction maps of SARS-CoV-2 proteins (cont'd)

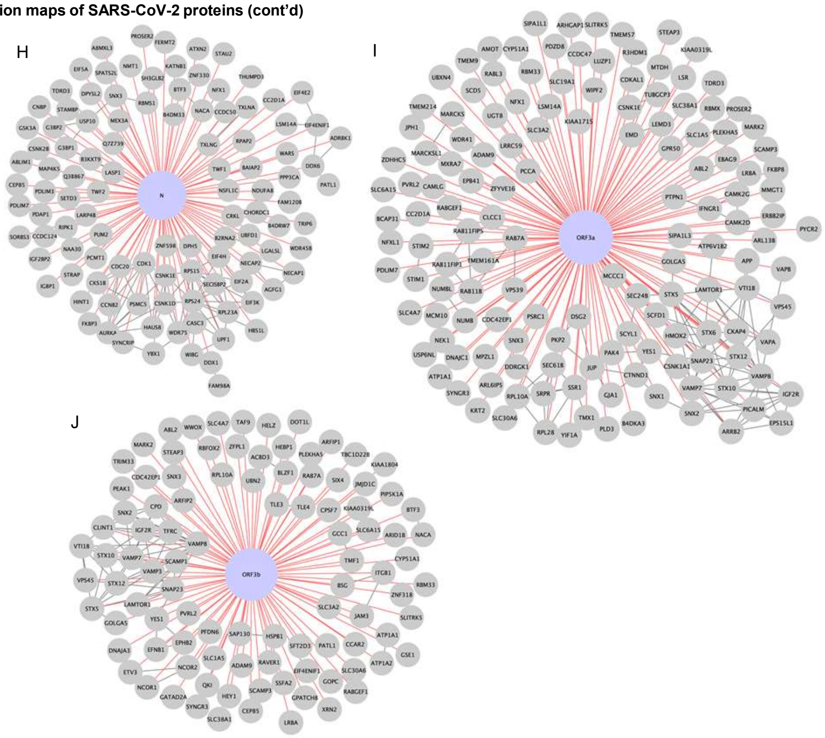

S2 Fig. Interaction maps of SARS-CoV-2 proteins (cont'd)

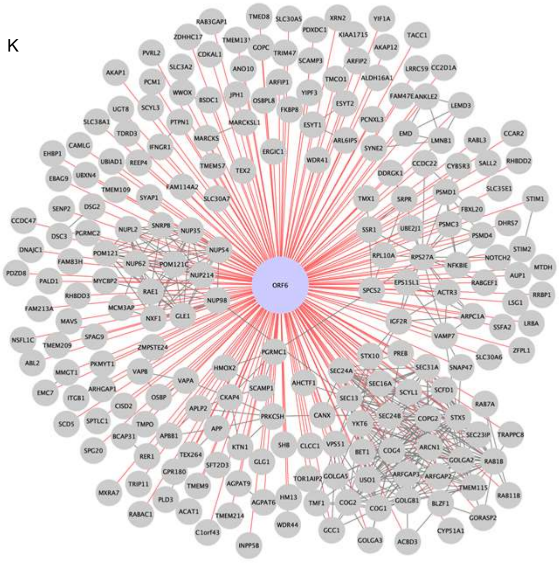

S2 Fig. Interaction maps of SARS-CoV-2 proteins (cont'd)

L

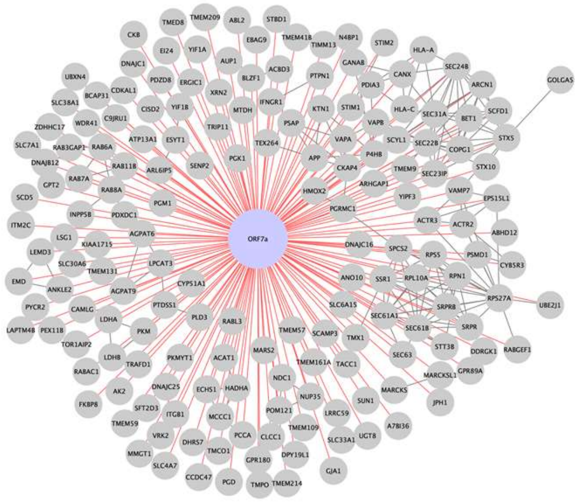

S2 Fig. Interaction maps of SARS-CoV-2 proteins (cont'd)

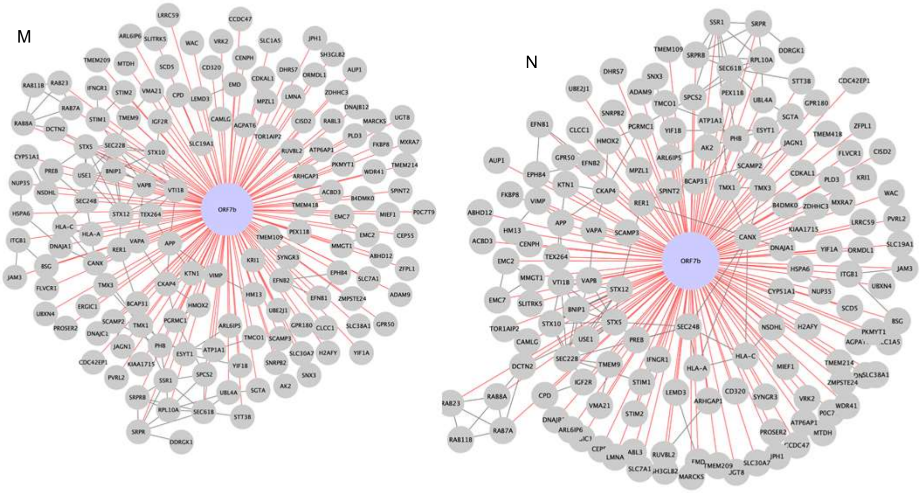

S2 Fig. Interaction maps of SARS-CoV-2 proteins (cont'd)

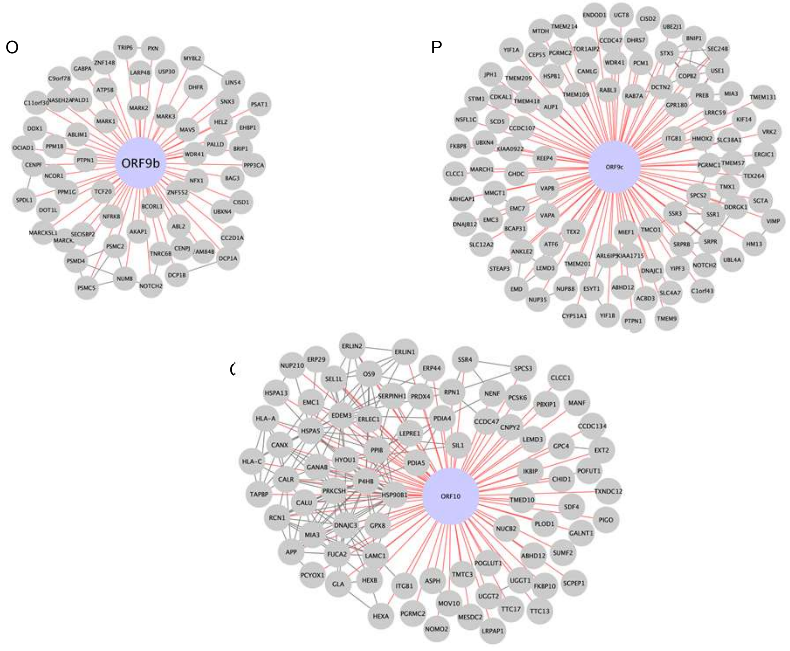

Supplement: S2 Fig — Interaction maps for all SARS-CoV-2 proteins(A-Q) from this study. All host proteins with SAINT scores ≥ 0.9 were included. (PDF) [file ppat.1009412.s002.pdf]
